# Controlling interferometric properties of nanoporous anodic aluminium oxide

**DOI:** 10.1186/1556-276X-7-88

**Published:** 2012-01-26

**Authors:** Tushar Kumeria, Dusan Losic

**Affiliations:** 1 Ian Wark Research Institute, University of South Australia, Mawson Lakes, Adelaide, South Australia, 5095, Australia

**Keywords:** nanoporous alumina, reflective interference spectroscopy, interference spectrum, optical label-free biosensing

## Abstract

A study of reflective interference spectroscopy [RIfS] properties of nanoporous anodic aluminium oxide [AAO] with the aim to develop a reliable substrate for label-free optical biosensing is presented. The influence of structural parameters of AAO including pore diameters, inter-pore distance, pore length, and surface modification by deposition of Au, Ag, Cr, Pt, Ni, and TiO_2 _on the RIfS signal (Fabry-Perot fringe) was explored. AAO with controlled pore dimensions was prepared by electrochemical anodization of aluminium using 0.3 M oxalic acid at different voltages (30 to 70 V) and anodization times (10 to 60 min). Results show the strong influence of pore structures and surface modifications on the interference signal and indicate the importance of optimisation of AAO pore structures for RIfS sensing. The pore length/pore diameter aspect ratio of AAO was identified as a suitable parameter to tune interferometric properties of AAO. Finally, the application of AAO with optimised pore structures for sensing of a surface binding reaction of alkanethiols (mercaptoundecanoic acid) on gold surface is demonstrated.

## Introduction

Label-free optical biosensing has attracted a considerable interest in recent years for biomedical and environmental applications regarding its simplicity, cost-effectiveness, easy miniaturisation, and superior performance [1,2]. In general, the principle is based on the detection of interfacial changes of the binding reaction at the surface and can be employed not only for a sensitive and selective measurement of specific biomolecules, but also for real-time monitoring of binding kinetics, thermodynamics, affinity, and specificity [3,4]. Label-free biosensing devices can incorporate different specific recognition elements, such as antibodies, DNA molecules, or enzymes that convert the reaction with a given analyte into a quantifiable signal such as an optical, acoustic, electrochemical, or mass change [2,5]. Among several optical methods based on surface plasmon resonance, enhanced Raman scattering, wave guiding, Bragg diffraction, and photonic bandgaps, the reflective interference spectroscopy [RIfS] method is recognised as particularly promising for the development of label- free biosensing devices [6-10].

The RIfS is a sensitive optical method based on white light interference at a thin film where the interference pattern depends on the product of the refractive index (*n*) and thickness (*L*) [11]. The binding of analyte to the surface of the thin film produces a change in the optical thickness (2*nL*) (i.e. product of film thickness and refractive index), and these changes result in a shift of the characteristic interference pattern measured in the optical spectrum. The RIfS methods using polymer films and membranes have been explored over the last two decades by the Gauglitz group for label-free detection of various molecules including proteins, DNA, herbicides, and hydrocarbons [4,6,11-14]. In addition to thin polymer films, the Sailor group has demonstrated that nanoporous structures such as porous silicon prepared by electrochemical etching could offer superior RIfS properties for chemical and biological sensing [15-19]. The ultimate advantage of a nanoporous RIfS platform is the provision of a three-dimensional structure with a large specific surface area for increased ligand immobilisation density and analyte capture in comparison with soft and chemically sensitive polymer films. The surface of porous silicon can also be easily modified with desired functional groups and covalently attached targeting biomolecules [20]. Therefore, it is not surprising that several groups have explored porous silicon as a promising optical interferometric biosensing platform for applications, including label-free sensing of DNA, antibodies, proteins, and cells [15-19,21-23]. However, these studies showed several limitations of porous silicon due to its poor stability and rapid degradation, which can adversely influence the biosensing signal [21]. To address this problem, new porous films were explored, with nanoporous anodic aluminium oxide [AAO] and titania nanotubes prepared by self-ordering electrochemical anodization of Al and Ti being recently introduced as new platforms for the development of more stable interferometric biosensing devices [24-27].

**Figure 1:**
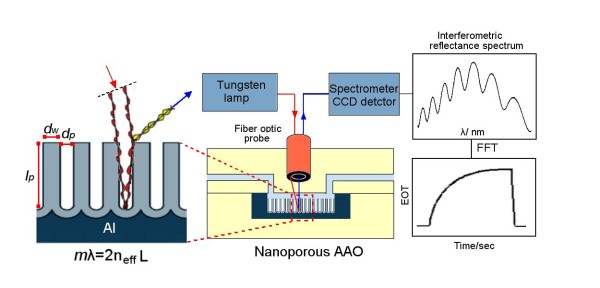
**Schematic diagram of the nanoporous AAO reflective interferometric sensor**. The basic optical setup includes an AAO platform placed into a microfluidic channel, fibre optic probe, light source, CCD detector, and computer. The principle of the interaction of light with AAO pores is presented on the left, and the generation of the interference pattern (Fabry-Perot fringes) processed by FFT analysis into a real signal is shown on the right.

Typically, the nanoporous AAO porous layer is composed of arrays of vertically aligned and highly organised (hexagonal pattern) pore structures [28]. AAO with controllable pore diameters ranging from 20 to 300 nm, inter-pore distances from 25 to 500 nm, and pore depths from 1 to > 200 μm were reported [28-31]. Derived from these properties, nanoporous AAO has found applications in many fields including molecular separations, adsorption, catalysis, biosensing, photonics, energy storage, fuel cells, nanofabrication, cell growth, and drug delivery [31-34]. AAO in comparison with porous silicon has greater pH, thermal stability, and fabrication flexibility to control pore structures, providing ultimate advantages for RIfS sensing [24]. A schematic of a RIfS interferometric sensor based on AAO is presented in Figure 1. It consists of an optical setup with a light source, AAO sensing platform placed into a microfluidic cell, charge-coupled device [CCD] spectrometer device for the detection of reflected optical signal, and PC for the quantification/presentation of signal by fast Fourier transform [FFT] analysis. Reflection of white light at the top and bottom of the AAO porous layer results in a characteristic interference pattern with Fabry-Perot fringes, which is dependent on the product of refractive index and thickness, and is sensitive to changes in the refractive index of the porous layer caused by the binding of analyte to the surface.

Similarly, to porous silicon, the interference signal which can be defined by the number of fringes and their intensity or amplitude from the porous AAO surface is assumed to depend on pore geometry, which suggests the importance of optimisation of AAO pore structures for RIfS sensing applications. To optimise the AAO platform, it is essential to have a better understanding of how pore structures influence RIfS properties. Hence, the aim of this work is to explore the impact of structural parameters of AAO on RIfS response with particular focus on pore diameters, inter-pore distance, pore wall thickness (defined by *d*_w _*= d*_i _*- d*_p_), pore length, and surface modification. Several examples are presented to show how the interferometric signal from AAO can be tuned by controlling pore dimensions using the anodization process (voltage and anodization time, pore widening, surface coating). Finally, a real-time measurement of binding alkanethiol molecules on gold surface using an AAO probe is demonstrated to confirm the capability of the AAO RIfS sensor in monitoring surface binding reactions.

## Experimental details

### Materials

Aluminium foil (thickness 0.1 mm, 99.997%) was supplied by Alfa Aesar (Ward Hill, MA, USA). Oxalic acid (Chem-Supply Pty Ltd, Adelaide, South Australia, Australia), chromium trioxide (Mallinckrodt Inc., Miami, FL, USA), phosphoric acid (85%, BDH, Poole, Dorset, UK), and mercaptoundecanoic acid [MUA] (Sigma-Aldrich Pty. Ltd, Castle Hill, New South Wales, Australia) were used as received. High-purity water was used for all solution preparations as produced by sequential treatments of reverse osmosis and a final filtering step through a 0.22-μm filter.

### Preparation of nanoporous AAO

Nanoporous AAO was prepared by a two-step anodization process using 0.3 M oxalic acid as an electrolyte at 0°C as previously described [28,31,35]. The first anodized layer of the porous alumina was prepared at a voltage of 60 to 80 V, then removed by an oxide removal solution (0.2 M chromium trioxide and 0.4 M phosphoric acid). For samples where the final anodization voltage was lower than the first anodization, an intermediate anodization step was carried out at 30 V for 60 min. Final anodization was carried out at a voltage varying between 30 and 70 V and for a time ranging from 2 min to 2 h in order to prepare AAO with desired pore diameters, inter-pore distances, and length. Pore-widening process in 10% phosphoric acid (10 min to 1 h) was performed to prepare AAO with equal inter-pore distances but different pore diameters.

### Structural characterisation of AAO

Pore diameters and thickness of the AAO film were determined using a scanning electron microscope [SEM] (XL 30, Philips, Amsterdam, The Netherlands). For cross-sectional SEM imaging, freestanding AAOs were prepared by removing the underlying Al using a solution (HCl + 0.2 M CuCl_2_). Prepared AAO samples for SEM characterisations were coated with a 3-nm Pt layer.

### Surface modification of prepared AAO

The coating of ultra-thin metal films (Au, Ag, Cr, Ni, and Pt) onto AAO was performed using metal vapour deposition by Emitech K975X (Quorum Technologies, Ashford, UK). The thickness of deposited films was approximately 8 nm and controlled by the film thickness monitor. Atomic layer deposition of titania oxide using titanium tetrachloride and water as a precursor at a deposition temperature of 200°C was preformed as described previously [36].

### Optical setup for reflective interference measurements

Optical RIfS measurements were performed using a microfibre optic spectrometer (Jaz, Ocean Optics, Inc., Dunedin, FL, USA). A bifurcated optical fibre with one of its trunk illuminated by a tungsten lamp carried the light to the probe, and the reflected light was collected by the same probe and fed to the other trunk of the optical fibre, which at the end, fed the reflected light to the spectrometer. The spot size of the light from the probe onto the AAO surface was kept around 2 mm in diameter, and all the reflective interference data were collected at a spectral range from 400 to 900 nm from the AAO film. Effective optical thickness [EOT] can be obtained by calculating the slope of a straight line fitting the graph of *m *vs. 1/*λ *using a peak finding algorithm or by applying FFT to the interference spectra. FFT from IGOR Pro (WaveMetrics, Inc., Potland, OR, USA) library was applied to finally obtain the EOT (2*n*_eff_*L *value in the Fabry-Perot interference fringe equation) [37].

### Functional characterisation using microchip RIfS AAO sensor

Au-modified AAO substrates were placed into a custom-designed microfluidic channel connected with the fibre optic probe and programmable syringe pump (New Era Pump Systems, Inc., Farmingdale, NY, USA). The adsorption of MUA on the AAO surface introduced into the microfluidic cell at concentrations from 0.01 to 1 mM was continuously monitored. Rinsing with a water solution for 5 min was performed between each concentration change.

## Results and discussion

### Controlling *d*_p _and *l*_p _of AAO

**Figure 2:**
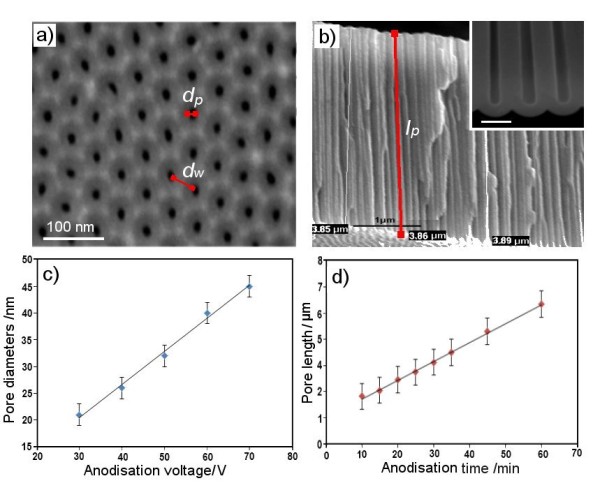
**Typical SEM images of nanoporous AAO prepared by Al anodization in 0.3 M oxalic acid**. (**a**) Top surface (50 V); (**b**) cross-sectional structure (50 V for 40 min); (**c**) graph showing the dependence of *d*_p _and anodization voltage (30 to 70 V); and (**d**) graph showing the dependence of *l*_p _and anodization time (from 10 to 60 min) under a constant anodization voltage (50 V).

Figure 2 shows SEM images of AAO routinely fabricated by anodization of Al in 0.3 M oxalic acid with typical pore morphologies observed from the top surface and in a cross-sectional view. Pore diameter [*d*_p_], pore wall thickness [*d*_w_], and pore length [*l*_p_] are considered as the three most important geometrical features of AAO that have a significant impact on the interaction with light and the RIfS signal. The top surface (Figure 2a) displays an array of self-ordered nanopores with a regular and highly organised hexagonal pattern confirming a typical AAO topography. The cross-sectional SEM images of the AAO film (Figure 2b) show that these pores are straight and vertically aligned with the bottom closed by a barrier oxide layer (inset). The underlying Al is typically not removed from the porous layer of AAO for RIfS studies, but was removed in this case for an imaging purpose. By controlling the anodization voltage from 30 to 70 V, we prepared AAO with a range of *d*_p _from 20 to 60 nm and inter-pore distances [*d*_i_] from 60 to 100 nm in order to study the influence of pore dimensions on the interference spectrum. The graph in Figure 2c confirmed a linear dependence of *d*_p _with the anodization voltage (30 to 70 V) which is in agreement with previous reports, showing the capability of this approach to control *d*_p _of AAO [29,31].

The thickness of the porous layer or *l*_p _was the second important parameter explored in this work, which is considered to have an influence on the RIfS signal. Because pore growth during the anodization process is a time-dependent process, anodization time is used as a simple strategy to control *l*_p _of AAO. However, the pore growth rate (in nanometers per minute) is not the same at different anodization voltages (30 to 70 V); thus, it is necessary to determine the growth rate for each of these conditions. The graph in Figure 2d presents an example of the *l*_p _(*l*_p _= 1.7 to 7 μm) prepared by anodization times (10 to 60 min) at 50 V. An average pore growth rate of 100 ± 35 nm/min was determined for this condition. When a higher anodization voltage (60 V) was applied, the rate of pore growth significantly increased (240 ± 25 nm/min). All other calibration graphs prepared using anodization voltages from 30 to 70 V showed a linear dependence between the *l*_p _and anodization time, confirming the ability of this approach to prepare AAO with controlled *l*_p_.

### The influence of *d*_p _on RIfS

**Figure 3:**
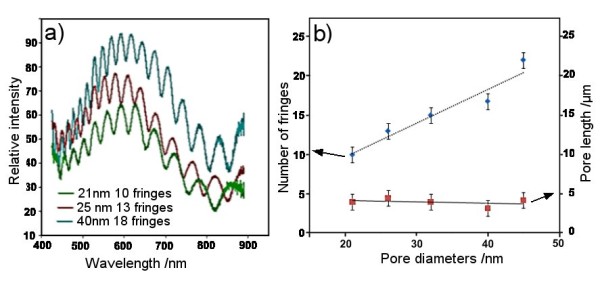
**Fabry-Perot fringe spectrum and relationship between *d*_p _and number of fringes**. (**a**) Fabry-Perot fringe spectrum obtained from AAO with *d*_p _of 21, 32, and 40 nm showing different numbers of fringes and (**b**) graph showing the relationship between *d*_p _and number of fringes. The *l*_p _was kept constant (approximately 4 μm).

Typical RIfS with Fabry-Perot fringes obtained from AAO with three different *d*_p _(21 ± 3, 25 ± 3, and 40 ± 2 nm) are presented in Figure 3a. To eliminate the effects of *l*_p_, the length for each AAO substrate was kept constant (*l*_p _= 4 μm). A significant increase in the number of fringes from 10 to 22 by increasing the *d*_p _is observed, showing a linear dependence (Figure 3b). The intensity of the interference signal was high and not considerably affected by changing the *d*_p _in this range. The number of fringes is increasing to more than 30 for diameters higher than 50 nm. The increased number of fringes due to *d*_p _can be explained by an increase in the number of light rays reflected at two interfaces that are in phase with each other. From an analytical point of view, the number of fringes is less critical than the intensity of the RIfS signal, and all these *d*_p _are suitable for RIfS measurements. However, the size of detecting molecules should also be considered during the selection of optimal *d*_p _as adsorption of small (< 1 nm) or large molecules (> 5 nm) inside of pores will have a different RIfS signal. A higher shift in the interference signal and a higher sensitivity is expected for the detection of small molecules in pores with smaller diameters than those with larger diameters.

**Figure 4:**
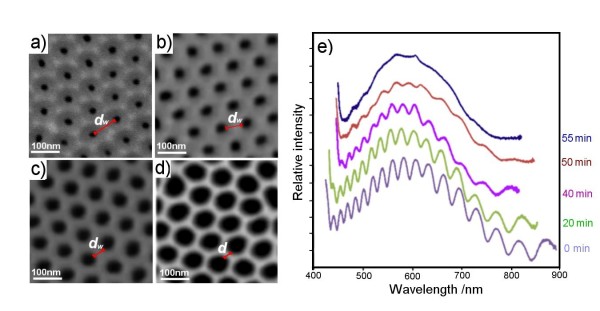
**SEM images of AAO pores and changes in the Fabry-Perot fringe spectrum of AAO**. SEM images of AAO pores with different *d*_w _prepared by the pore-widening process in 10 wt.% phosphoric acid from 0 to 55 min: (**a**) 0, (**b**) 20, (**c**) 40, and (**d**) 50 min. The reduction of pore walls is marked by a red line. (**e**) Changes in the Fabry-Perot fringe spectrum of AAO observed during the pore-widening process (initial *d*_p _= 30 nm).

Unfortunately, fabrication of AAO by controlling the anodization voltage not only produces AAO with different *d*_p_, but also with different *d*_i _and *d*_w_; thus, it is difficult to precisely elucidate their separate influence on the RIfS signal. To prepare AAO with different *d*_w _but the same *d*_i_, we applied a pore chemical dissolution process in 10 wt.% phosphoric acid called pore widening. The dissolution process of pores is expected to decrease the *d*_w _but also increase the *d*_p_. This is confirmed by SEM images (Figure 4a) showing a significant increase in *d*_p _from 30 to 70 nm and reduction of the *d*_w _from 70 (0 min) to 20 nm (50 min). The pore walls were completely dissolved (*d*_w _= 0 nm) after 65 min of the dissolution process (images not shown). Figure 4b presents changes in the interference fringe spectrum obtained from these samples, showing a continuous decrease in the number of fringes and decrease of their intensity with dissolution time.

The fringes completely disappeared from AAO treated by 55 min in phosphoric acid. The conclusion is clear: a smaller surface area between pores will provide a lower-intensity RIfS signal. This result is in apparent disagreement with previously presented results, where increased numbers of fringes were observed by increasing *d*_p_. It clearly indicates the importance of the *d*_w _as another parameter for optimisation of AAO platform for interferometric sensing. The reduced light reflection from AAO as a result of a decrease of the surface area between pores is the explanation for the observed impact on the RIfS signal. In the case of sensing applications based on surface binding detection, the availability of an active surface area on the top of pores vs. a surface area inside of pores could also have an impact on the sensitivity of the RIfS device.

### Impact of *l*_p_

**Figure 5:**
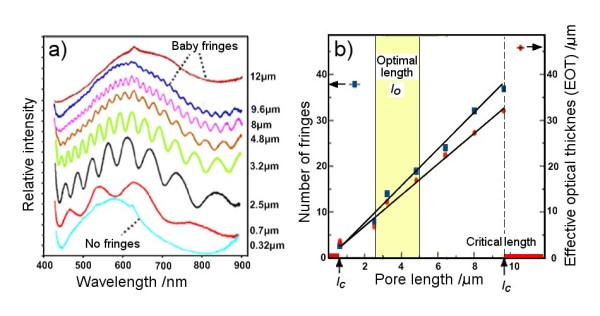
**Influence of *l*_p _on fringe spectrum and dependence of fringes and EOT with *l*_p_**. Influence of AAO *l*_p _on the Fabry-Perot fringe spectrum. (**a**) Series of interference spectrums obtained from AAO with *l*_p _from *l*_p _*= *0.3 μm to *l*_p _= 12 μm (*d*_p _= 40 nm were kept constant). No fringes were observed below *lp *= 0.3 μm, and baby fringes were observed above 9.6 μm. (**b**) Graph showing dependence of the number of fringes and EOT with *l*_p_. Regions with *l*_o _(2.5 μm > *l*_o _< 5 μm) and *l*_c _(0.3 μm <*l*_c _> 9.6 um) are marked in the graph.

Figure 5a presents changes in interference fringe patterns obtained from AAO with different *l*_p _from 300 nm to 12 μm, showing an increasing number of fringes with an increase in *l*_p_. Interestingly, in *l*_p _< 2.5 μm, only a few or no fringes are observed, and the most prominent increase in numbers occurs from *l*_p _> 2.5 μm to *l*_p _< 5 μm, showing fringes with a higher intensity (amplitude). The number of fringes continued to increase by increasing the *l*_p _from *l*_p _> 5 μm to *l*_p _= 10 μm, but with a significant decrease in their intensity. Interference spectrums with more than 30 fringes were observed, but the intensity of the fringe signal was too low and not useful for analytical measurements. By further increasing *l*_p _(*l*_p _> 10 μm), these fringes become very small and indistinguishable ('baby fringes'), and finally, interference signals were transformed into a fringeless spectrum (*l*_p _> 12 μm). These changes in the number of fringes and their intensity due to increasing *l*_p _are explained by the increased number of reflected light inside of pores and the simultaneous decrease in light intensity governed by multiple reflections (multiple trapping) of the light.

The influence of AAO *l*_p _on the number of fringes and EOT is summarised in Figure 5b. These graphs reveal two key parameters we called as critical [*l*_c_] and optimal [*l*_o_] pore lengths, which are important for optimisation of AAO RIfS properties. The *l*_c _is defined as the length of AAO when generated fringes become very small (baby fringe) or indistinguishable from the fringe spectrum. These fringe signals and AAO samples are not useful for RIfS measurements. Table 1 presents values of *l*_c _for five AAOs with different *d*_p _(20 to 40 nm) showing their dependence on *d*_p_. AAO with smaller *d*_p _= 20 nm showed significantly lower values of *l*_c _= 5.4 ± 0.5 μm than AAO with larger *d*_p _= 40 nm with *l*_c _= 9.5 ± 0.3 μm.

**Table 1:** The *l*_c _for AAO with *d*_p _from 20 to 40 nm

Pore diameter (nm)	20	24	32	40
Critical pore length (μm)	5.4 ± 0.5	7.3 ± 0.3	8.1 ± 0.2	9.5 ± 0.3

The *l*_o _are defined as the range, which provides both the highest intensity of RIfS signal and an optimal number of fringes. This value is also dependent on *d*_p _and requires specific optimisation for each diameter of AAO. The *l*_o _for AAO with a *d*_p _of 40 is in the range of *l*_p _> 2.5 μm to *l*_p _< 5 μm and results in an optimal number of fringes and intensity of the fringe signal.

**Figure 6:**
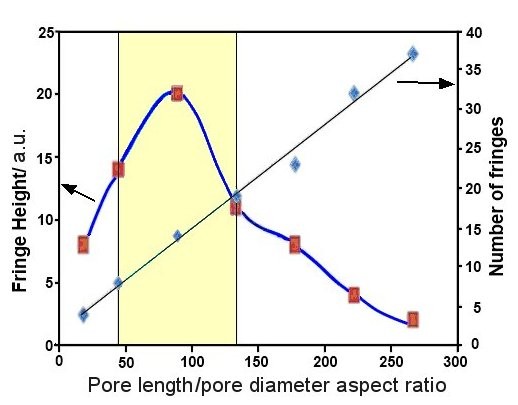
**Optimal *l*_p_*/d*_p _of AAO, which provides the highest interference signal**. The optimal number of fringes is presented by the highlighted yellow area (AAO prepared by 50 V).

From the presented results, we can conclude that the RIfS spectrum from AAO is dependent on several structural features including *d*_p_, *d*_w_, and *l*_p_, but importantly, their impact is combined. Hence, to design AAO with optimal RIfS properties for sensing applications, it is necessary to consider their collective rather than individual contribution. However, their combined influence on RIfS properties is very complex and difficult to use for practical application and fabrication of AAO. To simplify these structural influences, we identify a single parameter called the pore length/pore diameter aspect ratio [*l*_p_*/d*_p_], which can be used for the preparation of AAO with an optimised RIfS signal. Figure 6 reviews the optimal range of parameters taking into account two characteristics of the RIfS signal: the number of fringes and the maximum height of the fringe signal. This graph reveals that the optimal *l*_p_*/d*_p _of AAO for RIfS measurement is between 50 and 130, which can be easily achieved with the appropriate anodization conditions, including defining the voltage and anodization time.

### Influence of surface modifications

The reflectance of light from the surface depends on the reflectivity of the surface, which is also assumed to have an influence on the interference signal from the AAO surface. Thus, the influence of surface modification of AAO pores on the intensity of the fringe signal was explored to further optimise their RIfS properties. Two approaches were explored to modify the AAO surface, including deposition of ultra-thin metal films on the top surface and conformal coating inside of pores by the oxide film using atomic layer deposition [ALD]. The ultra-thin metal coatings were typically applied at an optimal thickness of approximately 7 to 8 nm, as thicker films could make substantial morphological changes to *d*_p _or alter and/or completely diminish the interference spectrum.

**Figure 7:**
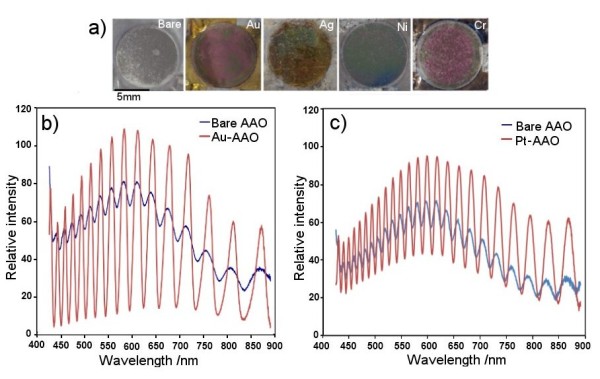
**Interference spectrum of modified AAO**. (**a**) Photos of AAO modified with different metal coatings. (**b**, **c**) Interference spectrum of AAO obtained before and after coating with thin Au and Pt layers (8 nm).

Photos of coated AAO samples with Pt, Au, Ag, Ni, and Cr reveal their different colours in comparison with unmodified AAO (Figure 7a). Table 2 summarises the results of the interference fringe intensity (maximum taken from the central fringe signal) obtained from these samples. The interference spectrums show that all metal coatings of AAO increased the intensity of the fringe signal in comparison with non-modified AAO. Interference spectrum obtained before and after coating with Au and Pt showed a typical change, with a significant increase in the intensity of the RIfS signal after coating (Figure 7b, c).

**Table 2:** Effect of different modifications of AAO pores on fringe intensity

Coating material	AAO	Pt	Au	Cr	Ni	Ag	TiO_2 _(ALD)
Thickness (nm)		8	8	9	5	13	10
Interference maximum intensity^a ^(a.u.)	10 ± 3	50 ± 5	98 ± 6	100 ± 8	65 ± 2	35 ± 2	11 ± 2

^a^Average of five measurements plus standard deviation. AAO, anodic aluminium oxide; ALD, atomic layer deposition; a.u., arbitrary unit.

The enhancement in intensity for the metal coating was about three to five times, with the highest signal for Au and Cr coatings. These changes in intensity are attributed to an increase in reflectivity of the coated AAO surface after thin-metal coating. Results were not surprising since it was expected that the deposition of reflective metal films will increase the intensity of the RIfS signal and therefore considerably improve optical properties of AAO. Additional advantages of the metal coating such as Au include its chemical stability and the ability to assemble desired molecules with a specific binding affinity toward targeting molecules important for biosensing applications. The RIfS signal from ALD conformal coating of AAO pores with a 10-nm-thick titania oxide layer did not show any enhancement in comparison to unmodified AAO. This result can be explained by the impact of this coating on the *l*_p_*/d*_p_, in addition to its lower reflectivity in comparison with metal films.

### Functional characterisation of AAO RIfS sensor

**Figure 8:**
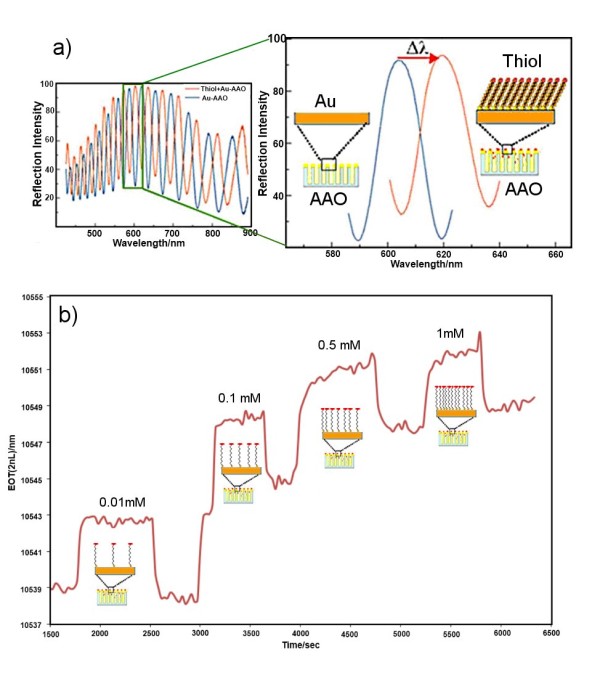
**Real-time measurement of molecular adsorption on the gold-coated AAO**. (**a**) Interference spectrum for the gold-coated AAO before and after exposure to 0.5 mM MUA with a single fringe signal representing a wavelength shift of interference maxima (on the right). (**b**) Changes of the EOT signal by continuous addition of different concentrations (0.01 to 1 mM) of MUA. The scheme of assembly of thiol molecules on gold surface is presented.

Finally, the sensing application of optimised AAO substrates with an *l*_p_*/d*_p _of 100 coated with gold film was demonstrated to sense surface binding reactions. A brief functional characterisation was performed for the adsorption of alkyl-thiol on gold as a model reaction using a microfluidic setup. The device was assembled by placing the AAO platform into a microfluidic chip connected with the pump and fibre optic probe. Figure 8a presents the typical RIfS signal before and after addition of 1 mM MUA into solution, showing that the wavelength of the maximum reflectance spectrum was shifted towards a longer wavelength as a result of adsorption of MUA molecules on the gold surface. A large shift in the interference signal (> 20 nm) is observed (Figure 8a) even when a very low concentration was used (< 10 nM), thus showing the excellent sensitivity of the AAO pore platform as an interferometric biosensing device. The shifting of the interference maxima after thiol exposure is a result of changes in the effective refractive index of the Au-coated AAO. As represented by a Fabry-Perot thin-film interference equation (*mλ *= 2*n*_eff_*L*), the wavelength of the *m*th maxima is in direct relation to the refractive index and film thickness; thus, any changes in the refractive index or thickness will be directly observed in the shifting of the *m*th maxima wavelength. The red shift in the maxima wavelength observed from the interference spectrum was resolved into EOT by applying FFT (Figure 8b) for real-time monitoring of the adsorption of thiol MUA on gold surface. A series of thiol solutions with concentrations from 0.01 to 1 mM were continuously added into the cell whilst recording the EOT signal.

At the low concentration of 0.01 mM, a big shift in EOT is observed, but after the introduction of water, the signal reaches again the baseline, indicating weak and reversible adsorption of thiol molecules. By increasing the thiol concentration, an increase in the EOT signal is observed, but without the return to baseline, showing an irreversible adsorption of the MUA. This result can be explained by the formation of more stable self-assembled monolayers due to an increasing thiol concentration. This brief study shows the capability of gold-modified AAO to be used as a sensitive device to detect surface-confined reactions.

## Conclusions

In summary, the evaluation of the RIfS performance of nanoporous AAO influenced by its structural and surface properties is presented. Parameters including *d*_p_, *d*_w_, *l*_p_, and surface modification were found to have a significant influence on the RIfS signal (number of Fabry-Perot fringes) and intensity of AAO. Tuning of these parameters by anodization conditions (voltage and anodization time) and additional chemical dissolution was demonstrated as an appropriate approach to control and optimise RIfS properties of AAO for sensing applications. However, our results showed that it is necessary to consider their collective rather than individual contribution, and the *l*_p_*/d*_p _is recommended as a simplified parameter for the optimisation process. Thin-metal coatings of AAO by different metals (Au, Cr, Ag, Pt, etc.) were found to considerably enhance the intensity of the interference spectrum (non-coated 8 to 15, coated 25 to 120). The ability to functionalise and attach sensing molecules onto these surfaces considerably extends the sensing capability of AAO as a RIfS platform. Finally, AAO with optimised pore dimensions coated with the gold film was characterised for adsorption of thiol molecules on gold, showing an excellent sensitivity to probe the surface binding reaction. In conclusion, these results confirm AAO as a promising RIfS platform for future generations of sensitive and selective label-free biosensing devices.

## Competing interests

The authors declare that they have no competing interests.

## Authors' contributions

TK carried out the experimental works, AAO preparation, surface modification, deposition, RIfS-based measurements, and SEM characterisation and drafted the manuscript. DL provided knowledge and supervisional support for this study and wrote the final version of the paper. All authors read and approved the final manuscript.
